# Protective effect of epigallocatechin-3-gallate (EGCG) via Nrf2 pathway against oxalate-induced epithelial mesenchymal transition (EMT) of renal tubular cells

**DOI:** 10.1038/srep30233

**Published:** 2016-07-25

**Authors:** Rattiyaporn Kanlaya, Supaporn Khamchun, Chompunoot Kapincharanon, Visith Thongboonkerd

**Affiliations:** 1 Medical Proteomics Unit, Office for Research and Development, Faculty of Medicine Siriraj Hospital, and Center for Research in Complex Systems Science, Mahidol University, Bangkok 10700, Thailand

## Abstract

This study evaluated effect of oxalate on epithelial mesenchymal transition (EMT) and potential anti-fibrotic property of epigallocatechin-3-gallate (EGCG). MDCK renal tubular cells were incubated with 0.5 mM sodium oxalate for 24-h with/without 1-h pretreatment with 25 μM EGCG. Microscopic examination, immunoblotting and immunofluorescence staining revealed that oxalate-treated cells gained mesenchymal phenotypes by fibroblast-like morphological change and increasing expression of vimentin and fibronectin, while levels of epithelial markers (E-cadherin, occludin, cytokeratin and ZO-1) were decreased. EGCG pretreatment could prevent all these changes and molecular mechanisms underlying the prevention by EGCG were most likely due to reduced production of intracellular ROS through activation of Nrf2 signaling and increased catalase anti-oxidant enzyme. Knockdown of Nrf2 by small interfering RNA (siRNA) abrogated all the effects of EGCG, confirming that the EGCG protection against oxalate-induced EMT was mediated via Nrf2. Taken together, our data indicate that oxalate turned on EMT of renal tubular cells that could be prevented by EGCG via Nrf2 pathway. These findings also shed light onto development of novel therapeutics or preventive strategies of renal fibrosis in the future.

One of the major causes of end-stage renal disease (ESRD) is unsolved chronic kidney disease (CKD) with fibrotic change. Renal fibrotic scar in the kidney in concert with several mediators and inflammatory response cause deteriorated kidney function^1^. Risk factors contributing to the development of fibrotic kidney include hypertension, diabetes mellitus, glomerulopathies, nephrotoxicity, etc.^1^,^2^. Recently, epithelial plasticity, also known as “epithelial mesenchymal transition” (EMT), has been found to be associated with renal fibrogenesis in adult kidney^3^,^4^,^5^,^6^. During embryonic phase, EMT is indeed the important process essential for normal development, in which anchored epithelial cells can be modified or rearranged to become an organ^7^. During pathogenic EMT, epithelial cell loses its epithelial phenotypes while gains the mesenchymal characteristics. Typical morphology of epithelial cell is changed into spindle-shape or fibroblast-like with a loss of the cell polarity, which is a characteristic of polarized epithelial cell^7^,^8^. In addition, epithelial markers, e.g. E-cadherin and tight junction (TJ) associated proteins (occludin, zonula occludens-1 or ZO-1), are down-regulated resulting to weakening of cell-cell adhesion/contact and paracellular/intercellular integrity. In contrast, mesenchymal markers, e.g. fibroblast-specific protein 1 (FSP1) and vimentin, are commonly up-regulated. In addition, overproduction of extracellular matrix proteins (e.g., fibronectin and collagen) and metalloproteases can be found during the EMT process^7^,^9^.

EMT can induce cytoskeletal reorganization and formation of actin stress fiber. FSP1 has been found in renal tubular cell after acute and chronic injury, suggesting that EMT is also involved in tissue repair process^10^. Interestingly, a previous study using a transgenic murine model has demonstrated that approximately one-third of renal interstitial fibroblasts were derived from tubular epithelial cells by EMT-dependent mechanism^11^. Consistently, a strong association between EMT and renal fibrosis has been confirmed in the study by Rastaldi *et al.*^12^. In this study, the investigators have demonstrated EMT features were correlated with degree of interstitial damage in 133 renal biopsies. In addition, EMT can be induced in renal epithelial cells derived from collecting duct by exposure to insulin-like growth factors (IGFs) and transforming growth factor-beta 1 (TGB-β1)^13^. Recently, an EMT marker Twist has been evidenced in renal biopsies from nephrolithiatic patients with large renal calculi^14^. Collectively, these lines of evidence have strongly indicated the association between EMT and renal fibrogenesis. Therefore, defining an inhibitor and its molecular mechanisms to suppress this process holds a great promise to pave the way for future treatment (hopefully recovery) of renal fibrosis.

More recently, *Camellia Sinensis* (green tea) has drawn lots of attention from scientists and clinicians because of its beneficial effects to prevent humans from lifestyle-related diseases^15^. Clinical investigations have demonstrated that green tea not only has anti-oxidative function but also offers anti-allergic, anti-carcinogenic, and anti-bacterial effects^16^,^17^,^18^,^19^. Among several polyphenols found in green tea extract, epigallocatechin-3-gallate (EGCG) is the major abundant catechin with a potent anti-oxidative property^20^,^21^. Interestingly, inhibitory effect of green tea on calcium oxalate (CaOx) crystallization has been demonstrated in animal models of kidney stone disease^22^,^23^. Its anti-oxidative property has been demonstrated in nephrolithiatic rat model induced by ethylene glycol^22^. Furthermore, the rats treated with green tea had decreased urinary oxalate excretion and diminished CaOx deposition in the kidney, while superoxide dismutase (SOD) activity was increased^23^. In addition to renal fibrosis, anti-fibrotic property of green tea has been demonstrated in experimental models of hepatic fibrosis^24^ and pulmonary fibrosis^25^.

Interestingly, histopathology shows lowered deposition of collagen in the kidney of animals treated with green tea^24^. In addition, administration of EGCG in a rat model of bleomycin-induced pulmonary fibrosis has demonstrated the involvement of nuclear factor erythroid-derived 2-related factor 2 (Nrf2) and Kelch-like ECH-associated protein 1 (Keap1) signaling. This pathway can enhance the anti-oxidative activity of phase II enzymes, including glutathione-S-transferase and NAD(P)H:quinineoxidoreductase 1 (NQO1), which can suppress inflammatory process. These findings suggest that green tea extract has combined beneficial effects on anti-inflammation, anti-oxidative stress and anti-fibrosis^25^.

Our group has recently reported the cellular adaptive responses of renal tubular epithelial cells in high-oxalate environment^26^. In this study, oxalate caused alterations in various biological processes associated with several cellular proteins, including those involved in stress response and actin cytoskeletal assembly^26^. In addition, global protein network analysis predicted one of the interacting proteins involved in Rho signaling. These findings prompt us to link between the oxidative stress and the induction of EMT by oxalate. The present study thus aimed to investigate EMT induction in renal tubular cells by oxalate and to examine protective effect of EGCG on such EMT induction, as well as molecular mechanisms underlying its anti-fibrotic property.

## Results

### Effect of EGCG on MDCK cell viability

**Figure 1:**
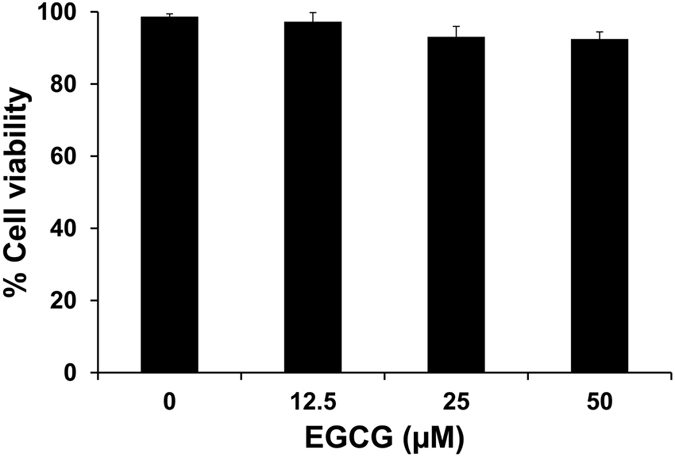
Effect of EGCG on MDCK cell viability. MDCK cells were treated with various doses of EGCG (0, 12.5, 25, and 50 μM) for 1 h and cell viability was determined by trypan blue exclusion assay. Statistical analysis of percentage of cell viability revealed no significant differences among different dosages used in this study.

Considering the potential cytotoxicity of EGCG in mammalian cells, MDCK cells were treated with various doses of EGCG for 1 h and cell viability was determined by trypan blue exclusion assay. The results showed that EGCG at 12.5–50 μM did not cause significant change in cell viability although it tended to be declined at a dosage of 50 μM (but did not reach statistical significant threshold) (Fig. 1). The mid-range dosage (25 μM) was then used for all subsequent experiments.

### Induction of EMT in MDCK cells by oxalate and protective effect of EGCG against oxalate-induced EMT

**Figure 2:**
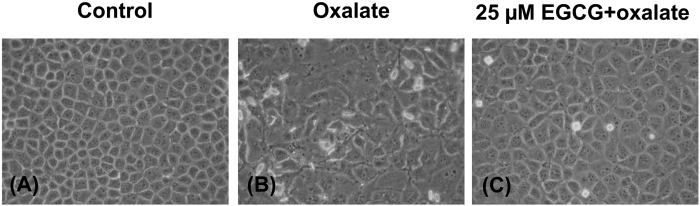
Induction of EMT in MDCK cells by oxalate and protective effect of EGCG against oxalate-induced EMT. Controlled or untreated MDCK cells (**A**) had typical cobblestone-like morphology, whereas the cells treated with 0.5 mM sodium oxalate (**B**) turned into fibroblast-like morphology within 24 h. Pretreatment with 25 μM EGCG for 1 h prior to sodium oxalate treatment (**C**) could prevent the cells from oxalate-induced fibroblast-like morphological change. Original magnification power = 400X.

After treatment with 0.5 mM sodium oxalate for 24 h, cell morphology was observed under a phase-contrast microscope. The result showed that morphology of oxalate-treated MDCK cells was changed from typical epithelial cells into elongated fibroblast-like cells as compared to those of the untreated (controlled) cells (Fig. 2A,B).

To evaluate the protective effect of EGCG against oxalate-induced EMT, MDCK cells were pretreated with 25 μM EGCG for 1 h prior to oxalate treatment as aforementioned. Microscopic examination revealed that epithelial cobblestone morphology was found in controlled cells and those pretreated with EGCG (Fig. 2A,C). Remarkably, the fibroblast-like morphology was not seen in the cells pretreated with EGCG followed by oxalate treatment (Fig. 2C).

### Examinations of epithelial and mesenchymal markers by Western blot analysis and immunofluorescence study

**Figure 3:**
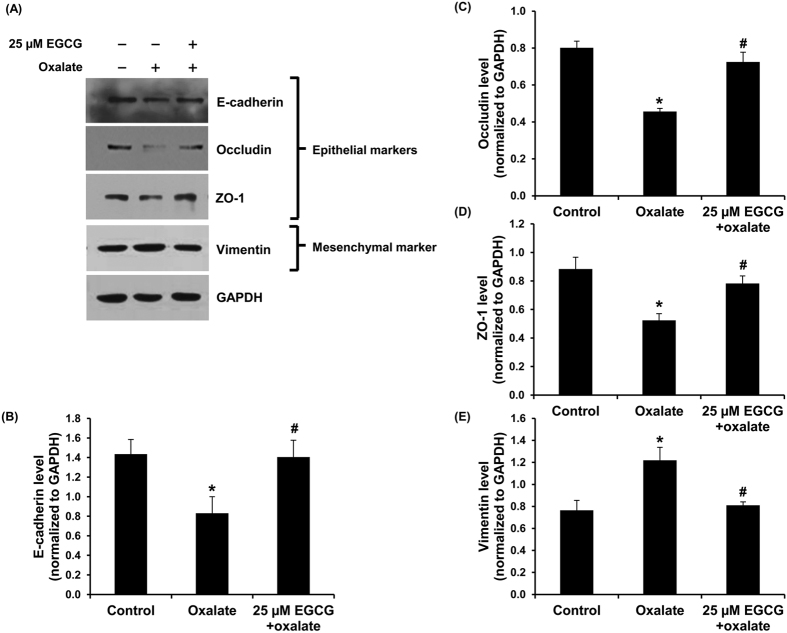
Western blot analysis of epithelial and mesenchymal markers. (**A**) Immunoreactive protein bands representing E-cadherin, occludin and ZO-1 (epithelial markers), and vimentin (mesenchymal marker) in different conditions. (**B**–**E**) Quantitative band intensity data revealed decreased levels of E-cadherin, occludin and ZO-1 and increased level of vimentin in oxalate-treated MDCK cells. Pretreatment with 25 μM EGCG for 1 h prior to sodium oxalate treatment could prevent the cells from such changes. GAPDH served as the loading control. N = 3 independent experiments. *p < 0.05 vs. control; ^#^*p* < 0.05 vs. oxalate group.

**Figure 4:**
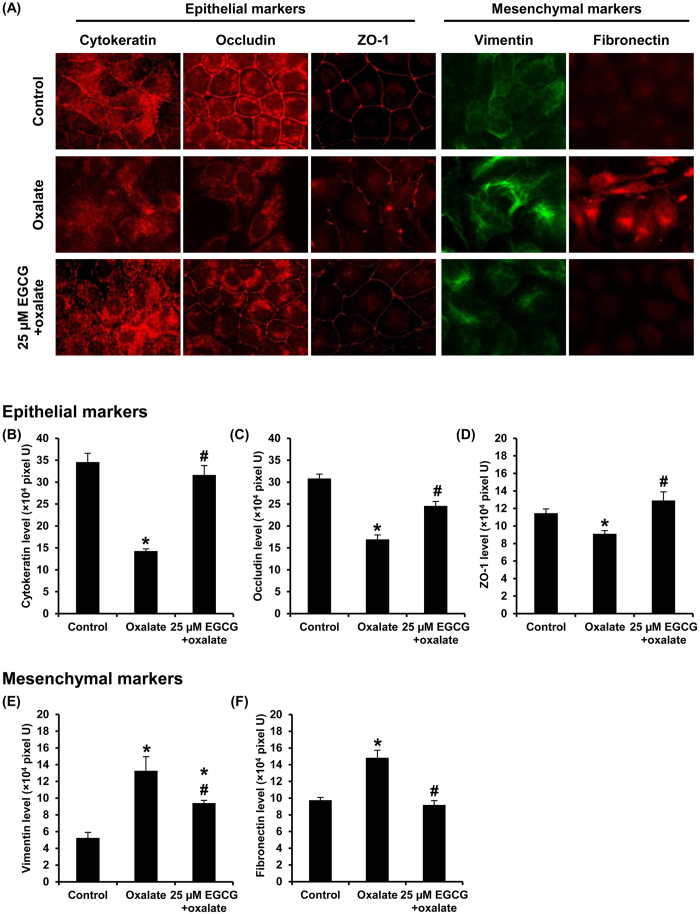
Examinations of epithelial and mesenchymal markers by immunofluorescence study. (**A**) Cytokeratin, occludin and ZO-1 (epithelial markers) were decreased, whereas vimentin and fibronectin (mesenchymal markers) were increased in oxalate-treated cells. Pretreatment with 25 μM EGCG for 1 h prior to sodium oxalate treatment could prevent the cells from such changes. Original magnification power = 1,000X. (**B**–**F**) Fluorescence intensity representing protein level was measured and analyzed from 10 random high-power fields (HPF) and at least 100 cells in each sample. N = 3 independent experiments. *p < 0.05 vs. control; ^#^*p* < 0.05 vs. oxalate group.

To further confirm that oxalate could induce EMT in renal tubular cells, Western blot analysis and immunofluorescence assay were performed to examine markers of epithelial (E-cadherin, occludin, cytokeratin, and ZO-1) and mesenchymal (vimentin and fibronectin) cellular phenotypes. The results by Western blot analysis revealed that oxalate significantly decreased expression of E-cadherin, occludin and ZO-1, but increased vimentin level. Pretreatment with EGCG could preserve these epithelial and mesenchymal markers at their basal levels (Fig. 3). Immunofluorescence study also revealed the decreased levels of epithelial markers (including cytokeratin, occludin and ZO-1) and increased levels of mesenchymal markers (vimentin and fibronectin) in oxalate-treated cells (Fig. 4). Moreover, the immunofluorescence data also showed that EGCG pretreatment successfully restored cytokeratin, occludin, ZO-1 and fibronectin to their basal levels, whereas the restoring effect on vimentin was partial (Fig. 4). Interestingly, there was a redistribution of the TJ proteins, occludin and ZO-1. These two TJ proteins were translocated from cell border into the cytoplasm in the oxalate-treated cells. However, EGCG pretreatment could successfully preserve these epithelial markers’ localizations and prevent their decrease induced by oxalate (Fig. 4A,C,D).

### Protective effect of EGCG was dose-dependent

**Figure 5:**
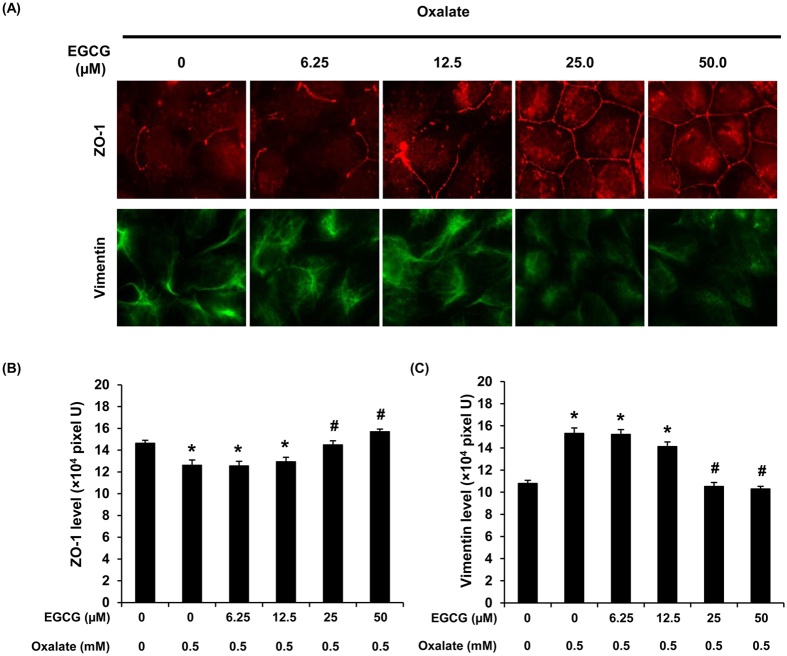
Protective effect of EGCG in oxalate-induced EMT was dose-dependent. (**A**) ZO-1 (a representative epithelial marker) and vimentin (a representative mesenchymal marker) were evaluated when the cells were pretreated with various doses of EGCG (0–50 μM) for 1 h prior to sodium oxalate treatment. Original magnification power = 1,000X. (**B**,**C**) Fluorescence intensity representing protein level was measured and analyzed from 10 random high-power fields (HPF) and at least 100 cells in each sample. N = 3 independent experiments. *p < 0.05 vs. control; ^#^*p* < 0.05 vs. oxalate group.

To investigate whether the protective effect of EGCG was dose-dependent, the cells were pretreated with various doses of EGCG (0–50 μM) for 1 h prior to oxalate treatment. The results by immunofluorescence study confirmed that EGCG had its protective role against oxalate-induced EMT (as demonstrated by the prevention against oxalate-induced decrease in ZO-1 and increase in vimentin expression levels) in a dose-dependent manner (Fig. 5).

### Oxalate-induced overproduction of reactive oxygen species (ROS) and reduction by EGCG

**Figure 6:**
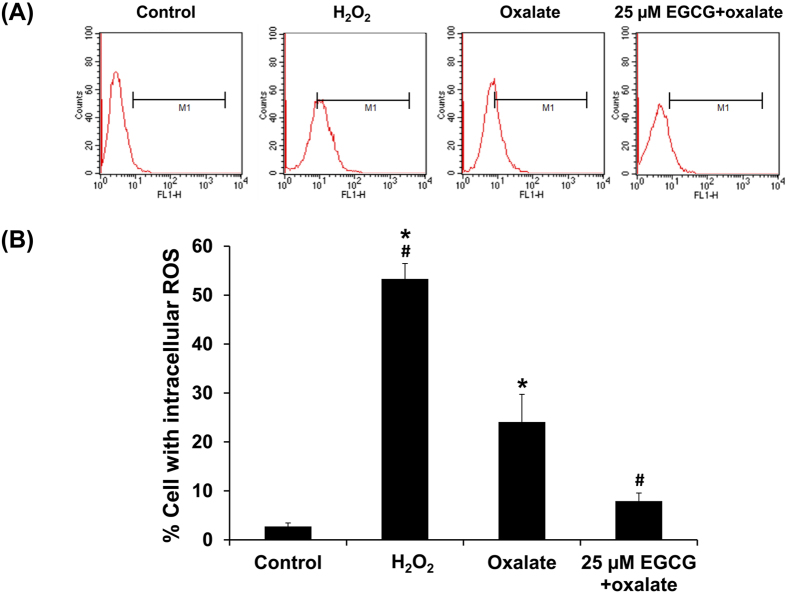
Oxalate-induced overproduction of reactive oxygen species (ROS) and reduction by EGCG. ROS production was evaluated by DCFH-DA assay and analyzed by flow cytometry. (**A**) Representative flow cytometric histograms and (**B**) Quantitative data of ROS production in different conditions. Untreated MDCK cells served as negative control, whereas those treated with hydrogen peroxide (H_2_O_2_) served as the positive control. The tested groups included those treated with 0.5 mM sodium oxalate for 24 h and those pretreated with 25 μM EGCG for 1 h prior to treatment with 0.5 mM sodium oxalate for 24 h. N = 3 independent experiments. *p < 0.05 vs. control; ^#^*p* < 0.05 vs. oxalate group.

As oxidative stress is one of the possible factors inducing EMT, we addressed whether the oxalate-induced EMT was also due to the increased intracellular production of ROS or not. As expected, the data using DCFH-DA assay and flow cytometry confirmed that percentage of intracellular ROS-positive cells was significantly increased (approximately 9-fold) in oxalate-induced EMT cells as compared to the controls (2.72 ± 0.72 vs. 24.07 ± 5.65% in controlled vs. oxalate-treated cells, respectively; p < 0.05) (Fig. 6). Interestingly, EGC pretreatment could dramatically reduce (although not completely abolished) the intracellular ROS production by approximately 2/3 (to remain only 1/3) as compared to oxalate-treated cells (24.07 ± 5.65 vs. 7.90 ± 1.67% in oxalate-treated vs. EGCG+oxalate-treated cells, respectively; p < 0.05) (Fig. 6). These results confirmed the potential role of oxidative stress in EMT induction by oxalate and demonstrated the potent anti-oxidant property of EGCG to prevent such induction.

### EGCG induced Nrf2 activation and intracellular catalase overproduction to prevent oxalate-induced EMT

**Figure 7:**
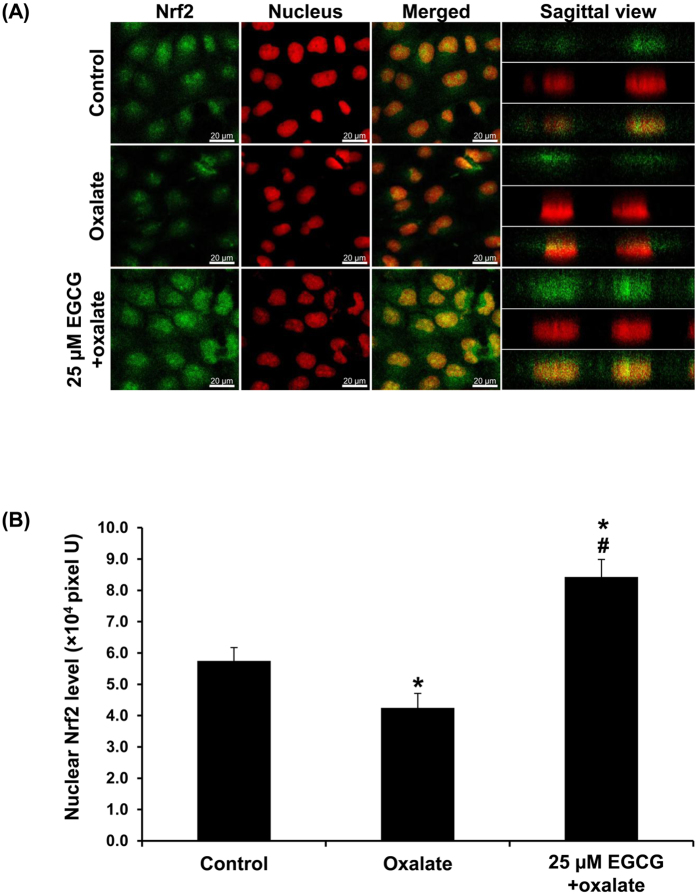
EGCG induced Nrf2 activation to prevent oxalate-induced EMT. (**A**) The cells were stained by indirect immunofluorescence assay using a specific antibody against Nrf2 (shown in green), whereas nuclei were counterstained with propidium iodide (shown in red). The images were captured under a laser-scanning confocal microscope. In merged fields and sagittal views, nuclear localization/translocation of Nrf2 is shown in yellow (in the nuclei). Original magnification power = 630X. (**B**) Fluorescence intensity representing Nrf2 level was measured and analyzed from 10 random high-power fields (HPF) and at least 100 cells in each sample. N = 3 independent experiments. *p < 0.05 vs. control; ^#^*p* < 0.05 vs. oxalate group.

In normal cellular response, chemicals-induced oxidative stress can be coped or controlled by activation of Nrf2 signaling pathway and overexpression of anti-oxidants. In this study, we thus performed indirect immunofluorescence study and laser-scanning confocal microscopic examination to determine the activation of Nrf2 by EGCG, which can be signified by its nuclear translocation. The results revealed that, in the controlled cells, Nrf2 expression was predominantly found in the cytoplasm with some degrees of nuclear localization (Fig. 7A). Oxalate treatment, however, lowered the overall expression of Nrf2 and its activated form as determined by lowered nuclear translocation of Nrf2. Pretreatment with EGCG dramatically increased Nrf2 expression in both cytoplasm and nuclei (Fig. 7A). Quantitative analysis of Nrf2 level showed that oxalate reduced Nrf, whereas EGCG pretreatment, on the other hand, increased Nrf level as compared to both controlled and oxalate-treated conditions (Fig. 7B).

**Figure 8:**
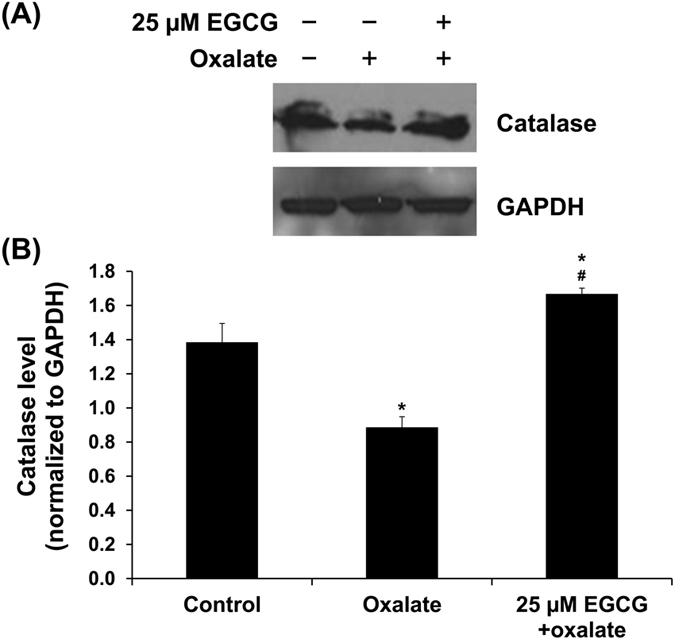
EGCG induced intracellular catalase overproduction to prevent oxalate-induced EMT. (**A**) Immunoreactive protein band representing catalase in different conditions. (**B**) Quantitative band intensity data revealed decreased level of catalase in oxalate-treated MDCK cells. Pretreatment with 25 μM EGCG for 1 h prior to sodium oxalate treatment markedly increased the level of catalase when compared to both controlled and oxalate-treated groups. GAPDH served as the loading control. N = 3 independent experiments. *p < 0.05 vs. control; ^#^*p* < 0.05 vs. oxalate group.

Since EGCG has been reported as a modulator or inducer of Nrf2-mediated anti-oxidant enzyme, we therefore investigated the intracellular level of catalase induced by Nrf2 signaling. Western blot analysis revealed that oxalate reduced intracellular anti-oxidant enzyme catalase, whereas EGCG pretreatment, vice versa, increased the catalase level as compared to both controlled and oxalate-treated conditions (Fig. 8), consistent to the data on Nrf2 level (Fig. 7). These results suggested that EGCG could induce Nrf2-mediated anti-oxidant enzyme production.

### Knockdown of Nrf2 by small interfering RNA (siRNA) abrogated protective effect of EGCG against oxalate-induced EMT

**Figure 9:**
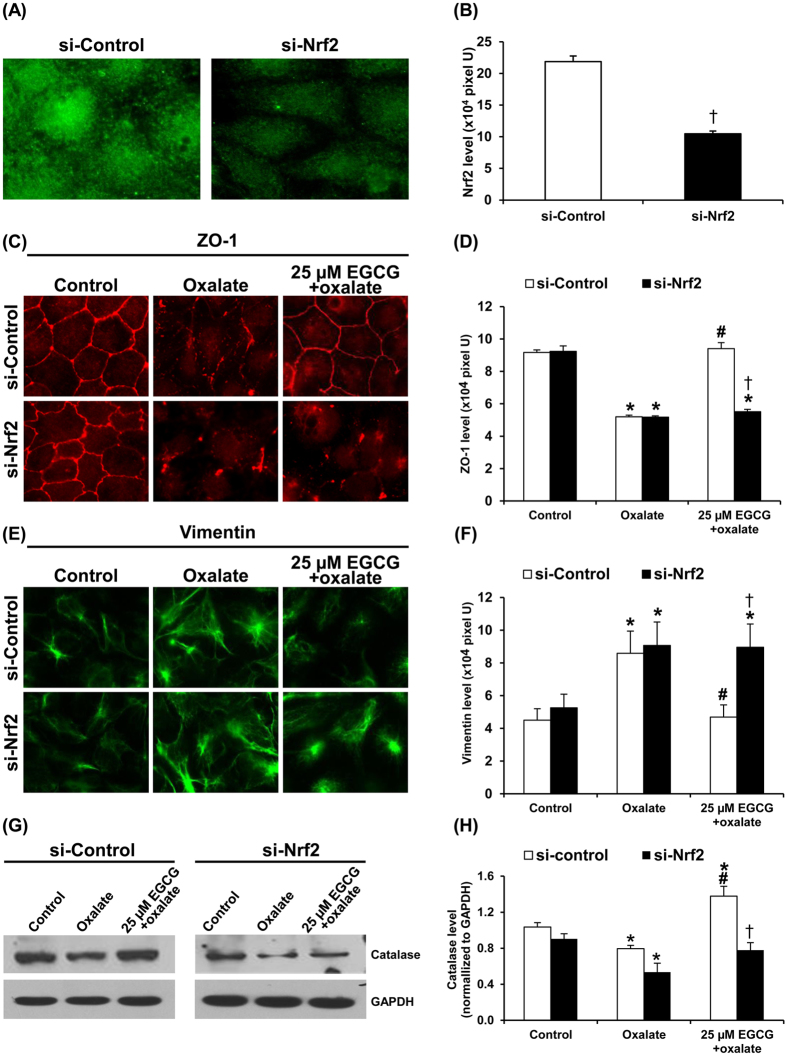
Si-Nrf2 abrogated the protective effect of EGCG against oxalate-induced EMT. (**A**,**B**) Efficacy of Nrf2 knockdown by si-Nrf2 was confirmed by immunofluorescence staining in the si-Nrf2-transfected cells, whereas the si-Control-transfected cells served as the control. (**C**,**D**) ZO-1 expression was evaluated as a representative epithelial marker. (**E**,**F**) Vimentin expression was evaluated as a representative mesenchymal marker. (**G**,**H**) Catalase, a product of Nrf2 activation, was evaluated by Western blotting (GAPDH served as the loading control). Original magnification power = 1,000X in all panels of (**A**,**C**,**E**). Fluorescence intensities representing Nrf2 (**B**), ZO-1 (**D**), and vimentin (**F**) levels were measured and analyzed from 10 random high-power fields (HPF) and at least 100 cells in each sample. N = 3 independent experiments. *p < 0.05 vs. control; ^#^*p* < 0.05 vs. oxalate; ^†^*p* < 0.05 vs. si-Control group.

To confirm that the protective effect of EGCG against oxalate-induced EMT was mediated via Nrf2 pathway, we performed Nrf2 knockdown of the cells using siRNA specific to Nrf2 (si-Nrf2), whereas the cells transfected with an equal dose of controlled siRNA (si-Control) served as the control. Efficacy of the Nrf2 knockdown was validated by immunofluorescence staining, which showed significantly decreased expression level of Nrf2 in the si-Nrf2-transfected cells as compared to the si-Control-transfected cells (Fig. 9A,B). While the si-Control-transfected cells exhibited all the effects of oxalate and EGCG identical to the non-transfected cells, the si-Nrf2-transfected cells showed that the protective effect of EGCG against oxalate-induced EMT was completely abolished as demonstrated by expression of epithelial (Fig. 9C,D) and mesenchymal (Fig. 9E,F) markers, as well as catalase level (Fig. 9G,H). These data confirmed that the protective effect of EGCG against oxalate-induced EMT was mediated via Nrf2 pathway.

## Discussion

In this study, we reported that oxalate could induce EMT in renal tubular epithelial cells. The concentration of sodium oxalate used in the present study was selected based on a previous study by Coe *et al.*^27^ reporting that approximately 0.5 mM oxalate is the maximal concentration of oxalate excreted into the urine by healthy individual. The dosage of EGCG was selected based on cell viability assay. Such dosage was in the middle range of the dosages tested in our present study that showed no obvious cytotoxicity induced by EGCG chemical exposure (Fig. 1). Induction of EMT by oxalate was supported by change in cell morphology from typical epithelial cell into fibroblast-like feature (Fig. 2). In addition to morphological change, oxalate-treated cells gained mesenchymal characteristics in which mesenchymal protein markers, including vimentin and fibronectin, were highly expressed in the oxalate-treated cells as compared to the controlled cells (Figs 3 and 4). In addition, loss of epithelial features was observed as demonstrated by lowered expression of E-cadherin, a hallmark of epithelial protein marker, and also decreased expression of tight junction (TJ) markers, occludin and ZO-1 (Figs 3 and 4). Moreover, redistribution of occludin and ZO-1 was obvious in oxalate-treated cells – they were almost absent from the TJ located at the cell borders and translocated into the cytoplasm, indicating disruption of cell-cell contact (Fig. 4). Interestingly, EGCG pretreatment could prevent all of these deteriorate effects of oxalate.

Several lines of evidence have revealed that oxalate could induce oxidative stress by increasing reactive oxygen species (ROS) production in renal tubular epithelial cells. Rashed *et al.*^28^ have demonstrated that peroxidative injury induced by oxalate was associated with an induction of TGF-β1 and imbalance of redox reaction by glutathione system. However, glutathione redox status and TGF-β1 were restored to their basal levels by treatment with anti-oxidants, including vitamin E and catalase^28^. Importantly, TGF-β1, a potent fibrotic factor that can also promote EMT, could trigger production of intracellular ROS in rat proximal tubular epithelial cells^29^ and MDCK cells^30^. From these studies, it has been hypothesized that ROS overproduction and alterations in redox homeostasis may potentially contribute to EMT progression.

Theoretically, EGCG with potent anti-oxidative activity might control the progression of EMT by serving as a ROS scavenger. However, there was no direct evidence to show that ROS production induced by oxalate could trigger the process of EMT and EGCG could inhibit such effects. In attempt to address this issue, we then measured intracellular ROS after oxalate treatment with or without EGCG pretreatment by pulsing with DCFH-DA and analyzing by a flow cytometer. As expected, the results clearly showed that intracellular ROS was significantly increased when the cells were induced by oxalate and underwent EMT comparing to the untreated controlled cells (Fig. 6). In addition, we showed that EGCG pretreatment could prevent the conversion of epithelial cells into fibroblast-like cells (Fig. 2) and the increase of intracellular ROS production by oxalate (Fig. 6). In consistent with the study of peritoneal fibrosis in a mouse model, EGCG could suppress NF-kB activation and ROS generation, and thus prevent those animals from peritoneal fibrosis^31^.

Furthermore, EGCG has been reported as an inducer of anti-oxidant enzymes (e.g. glutathione-S-transferase, glutathione peroxidase, superoxide dismutase, hemeoxygenase 1, catalase, etc.). The key mechanism of EGCG-induced anti-oxidant enzyme production has been reported to be related with activation of nuclear factor erythroid-derived 2-related factor 2 (Nrf2) signaling^32^. Nrf2 is an important transcription factor that plays a central role in cytoprotection by maintaining cellular homeostasis through anti-oxidant enzyme expression and xenobiotic transporters^33^. Under normal or physiologic condition, Nrf2 is within a complex with Kelch-like ECH-associated protein 1 (Keap 1), which in turn facilitates the degradation of Nrf2 by proteasome^33^. Based on our present data, the mechanism by which EGCG induced Nrf2 has been proposed to occur via conjugation of reactive forms of EGCG with glutathione (GSH). This could lower cellular GSH level, thereby transiently disturbed redox status, resulting in activation of mitogen-activated protein kinases (MAPKs) (e.g. c-Jun, JNK, PI3K, ERK) and subsequent phosphorylation and activation of Nrf2^32^,^34^,^35^. Alternatively, some reactive (or electrophilic) forms of EGCG might interact with cysteine residues present in Keap1, leading to conformational change and dissociation of Keap1-Nrf2 complex^32^,^35^,^36^. Activation of Nrf2 by EGCG could ultimately lead to increased levels of detoxifying or anti-oxidant enzymes through association between Nrf2 and anti-oxidant-response element (ARE).

Interestingly, the renoprotective role of activated Nrf2 has been demonstrated in several previous studies. Shin *et al.*^37^ have shown that triggering of Nrf2-heme oxygenase-1 (HO-1) system could protect cyclosporin A-induced renal fibrosis by modulating EMT genes. Kang *et al.*^38^ have illustrated that angiotensin II-induced EMT could reduce levels of several Nrf2-dependent anti-oxidant enzymes and might contribute to progression of renal fibrosis. Hence, targeting Nrf2 using a powerful activator would provide a promising strategy to improve the treatment of CKD induced by sustained oxidative stress and inflammation^39^.

Therefore, we also hypothesized that EGCG might negatively regulate EMT via modulating Nrf2 pathway. Our results revealed that pretreatment with EGCG could induce Nrf2 activation in MDCK cells treated with oxalate as demonstrated by increasing expression and nuclear translocation of Nrf2 (Fig. 7). However, it was unexpected that treatment with oxalate alone resulted in decreased Nrf2 and its activation. Basically, under stress condition, cellular defense mechanisms are triggered in order to cope with oxidative damage. It might be possible that the cells were struggled by the sustained high oxalate exposure and could not overcome such extreme condition that ultimately led to the decreased Nrf2 activation. However, our findings were consistent to those reported by a recent study demonstrating that intraperitoneal administration of EGCG in rats with unilateral ureteral obstruction (UUO) efficiently suppressed oxidative stress induced by acute kidney injury (AKI)^40^. This inhibitory effect was mediated by its anti-oxidative property that might be partially activated by Nrf2 signaling pathway because the EGCG-treated group showed the increased nuclear Nrf2 expression along with the improved renal ultrastructure^40^.

In another study on bleomycin-induced pulmonary fibrosis, EGCG exerted roles in Nrf2-Keap1 signaling through enhancing anti-oxidant enzyme activities and inhibition of inflammation^25^. Similarly, the study on cisplatin-induced nephrotoxicity in rats has shown that EGCG administration could restore anti-oxidant enzyme activities, including catalase, through Nrf2 activation and attenuate kidney injury and inflammation^41^. We thus examined level of anti-oxidant enzyme catalase in our present study. Our data on catalase (Fig. 8) was in concordance with the expression and activation of Nrf2. Catalase level was significantly increased when the cells were pretreated with EGCG prior to oxalate exposure. The increased catalase level was expected to be a cellular defense mechanism mediated by EGCG-induced Nrf2 pathway in order to restore the cells to a basal state. Our data also supported a major role of catalase in the defense against oxidative tissue injury and renal interstitial fibrosis observed in the catalase-deficient mice^42^. Finally, we confirmed that the protective effect of EGCG was specifically mediated via Nrf2 signaling by evaluation of expression of epithelial and mesenchymal markers as well as level of catalase (one of the Nrf2 downstream products) in the si-Nrf2-transfected cells compared to the si-Control-transfected cells. Our data nicely confirmed that the molecular mechanisms of EGCG in prevention of oxalate-induced EMT were mediated via Nrf2 pathway (Fig. 9).

In summary, the present study has shown that oxalate could induce EMT of renal tubular cells. We also demonstrated that pretreatment with EGCG prior to oxalate induction successfully prevented oxalate-induced deteriorated changes by restoring epithelial phenotypes/markers and abolishing mesenchymal phenotypes/markers. Molecular mechanisms underlying the protective effect of EGCG were mediated through its anti-oxidant property by activation of Nrf2 signaling pathway, resulting to the increased level of catalase to cope with intracellular ROS overproduction. Our findings provided another piece of evidence that oxidative stress is one among various factors driving epithelial cells to EMT. It should be noted that high oxalate-induced EMT may be found in patients with hyperoxaluria and may increase a risk of developing renal fibrosis. Our data also implicated that EGCG may potentially be useful as an anti-fibrotic molecule for hindering EMT progression and may be of great value to patients with renal fibrosis in the future.

## Materials and Methods

### Cell culture

Mardin-Darby Canine Kidney (MDCK), a distal renal tubular epithelial cell line^43^,^44^ was cultivated in the growth medium containing Eagle’s minimum essential medium (MEM) (Gibco; Grand Island, NY) supplemented with 10% heat-inactivated fetal bovine serum (FBS) (Gibco) in the presence of 100 U/ml penicillin G and 100 mg/ml streptomycin (Sigma; St. Louis, MO). Cells were maintained in a humidified incubator at 37 °C with 5% CO_2_.

### Cytotoxicity test of EGCG

MDCK cells were seeded in 24-well plate in the growth medium 24 h prior to treatment. The cells were then incubated with 0, 12.5, 25 and 50 μM EGCG (Sigma) for 1 h. Thereafter, the cells were detached from the cultured well using 0.1% trypsin in 2.5 mM EDTA and immediately resuspended in MEM supplemented with 10% FBS to terminate trypsin activity. Aliquots of cell suspension were mixed with 0.4% trypan blue solution (Gibco) and the cells were then counted using a hemacytometer. Percentage of trypan blue-negative cells representing viable cells is reported.

### Induction of epithelial mesenchymal transition (EMT) by oxalate

MDCK cells were seeded at a density of 4 × 10^4^ cells/well and maintained in the growth medium for 24 h. Thereafter, the cells were washed with serum-free medium and then treated with 0.5 mM sodium oxalate (Sigma) in a maintenance medium (MEM supplemented with 1% heat-inactivated FBS) for 24 h. The cells without any treatment served as the control.

### Prevention of EMT by pretreatment with epigallocatechin-3-gallate (EGCG)

MDCK cells were seeded at a density of 4 × 10^4^ cells/well and maintained in the growth medium for 24 h. Thereafter, the cells were washed with serum-free medium and then pretreated with 25 μM EGCG in the maintenance medium (MEM supplemented with 1% heat-inactivated FBS) for 1 h (Note that 0, 6.25, 12.5, 25, and 50 μM EGCG were used at this step for the dose-dependent study). Thereafter, the cells were treated with 0.5 mM sodium oxalate in the maintenance medium for 24 h. The cells without any treatment served as the control.

### Morphological study and immunofluorescence staining of epithelial and mesenchymal markers

Cell morphology was observed under a phase contrast microscope (Olympus CKX41; Tokyo, Japan). For indirect immunofluorescence assay, the cells were grown on coverslip and EMT was induced as described earlier. After washing with PBS, the cells were fixed with 3.7% (v/v) formaldehyde in PBS for 10 min and then permeabilized with 0.2% Triton X-100 in PBS for 10 min. After washing, the cells were incubated with each of primary antibodies, including mouse monoclonal anti-vimentin conjugated with Alexa Fluor 488 (Invitrogen; Eugene, OR), mouse monoclonal anti-fibronectin (Santa Cruz Biotechnology; Santa Cruz, CA), mouse monoclonal anti-cytokeratin (Santa Cruz Biotechnology), rabbit polyclonal anti-occludin (Santa Cruz Biotechnology), and mouse monoclonal anti-ZO1 (Invitrogen) at 4 °C overnight (all were diluted 1:50 in 1%BSA/PBS). Except for vimentin staining, the cells were further incubated with corresponding secondary antibody conjugated with Cy3 (Dako; Glostrup, Denmark) at a dilution of 1:2,000 in 1%BSA/PBS at room temperature (RT) (set at 37 °C) for 1 h. Thereafter, the cells were extensively washed with PBS and mounted onto a glass slide using ProLong Gold anti-fade reagent (Invitrogen). The images were captured under the Nikon Eclipse 80i fluorescence microscope (Nikon; Tokyo, Japan). Mean fluorescence intensity representing protein level was analyzed from 10 random high-power fields (at least 100 cells in each sample) using NIS-Elements D V.4.11 (Nikon, Tokyo, Japan).

### Western blot analyses

Proteins were extracted from individual samples using Laemmli’s buffer and protein concentrations were measured by Bradford’s method using Bio-Rad Protein Assay (Bio-Rad Laboratories; Hercules, CA). Equal amount of total protein (30 μg/lane) from each sample was separated by 12% SDS-PAGE and transferred onto a nitrocellulose membrane. After blocking non-specific bindings with 5% skim-milk/PBS for 1 h, the membrane was incubated with mouse monoclonal anti-vimentin, rat monoclonal anti-E-cadherin, rabbit polyclonal anti-occludin, rabbit polyclonal anti-ZO-1, rabbit polyclonal anti-catalase, or mouse monoclonal anti-GAPDH antibody (all were purchased from Santa Cruz Biotechnology and diluted 1:1,000 in 1% skim milk/PBS) at 4 °C overnight. After probing with corresponding secondary antibody conjugated with horseradish peroxidase (Dako) at a dilution of 1:2,000 in 1% skim milk/PBS at RT for 1 h, the immunoreactive protein bands were visualized by SuperSignal West Pico chemiluminescence substrate (Pierce Biotechnology, Inc.; Rockford, IL) and autoradiography. Band intensity data was obtained using ImageQuant TL software (GE Healthcare; Uppsala, Sweden).

### Analysis of intracellular reactive oxygen species (ROS) production by dichlorofluoresceindiacetate (DCFH-DA) assay and flow cytometry

Intracellular ROS production was analyzed by DCFH-DA assay and flow cytometry. Briefly, the cells treated with oxalate alone or EGCG+oxalate were collected as suspension by trypsinization and incubated with 50 μM dichlorofluoresceindiacetate (DCFH-DA) (Invitrogen) for 30 min. The cells were then kept on ice and immediately analyzed by FACScan equipped with CellQuest software (Benton Dickinson; Franklin Lake, NJ). The cells left untreated were used as the negative control to determine basal intracellular ROS level, whereas those treated 0.02% (v/v) H_2_O_2_ served as the positive control.

### Immunofluorescence staining of Nrf2 and laser-scanning confocal microscopy

Activation of Nrf2 was illustrated by translocation of Nrf2from cytoplasm into the nucleus by indirect immunofluorescence assay and laser-scanning confocal microscopy. The cells were grown on a coverslip and treated with oxalate with or without EGCG pretreatment as aforementioned. The cells were then fixed and permeabilized simultaneously by incubation in ice-cold methanol for 10 min. After washing with PBS, the cells were incubated with mouse monoclonal anti-Nrf antibody (Santa Cruz Biotechnology) at a dilution of 1:50 in 1%BSA/PBS at 4 °C overnight. Thereafter, the cells were incubated with goat-anti-mouse-Ig G conjugated with Alexa Fluor 488 antibody (Invitrogen) at a dilution of 1:1,000 in 1%BSA/PBS at RT for 2 h. Nuclei were counterstained with 0.25 μg/ml propidium iodide (BD Bioscience; San Jose, CA). Finally, the coverslip was mounted onto a slide using 50% glycerol/PBS. 3-D planes of X-Y, X-Z and Y-Z scanning were captured under a laser-scanning confocal microscope (A1R, Nikon; Tokyo, Japan) equipped with NIS-Elements D V.4.11 (Nikon). The top view images were captured at nuclear cross-section. In addition, the cross-sectional signal was also recorded along its Z-axis (50 slices, 25 μM/each at Z-axis) to provide a data in sagittal view. The region of interest (ROI), which was the area in nuclei with yellow color in merged panel (representing nuclear Nrf2), was drawn for measuring fluorescence intensity. Quantitative data was analyzed from 10 random high-power fields (HPF) and at least 100 cells in each sample using NIS-Elements D V.4.11 (Nikon).

### Nrf2 knockdown

The small interfering RNA (siRNA) duplexes against Nrf2 (si-Nrf2) (Santa Cruz Biotechnology) and control siRNA (si-Control) (Santa Cruz Biotechnology) were transfected to MDCK cells. Briefly, the cells seeded in 6-well plate were transfected with 80 pmol si-Nrf2 or si-Control mixed with siRNA transfection reagent in the transfection medium (Santa Cruz Biotechnology) according the manufacturer’s instructions. The transfected cells were then incubated in a humidified incubator with 5% CO_2_ at 37 °C for 6 h. After 24-h post-transfection, the cells were subjected to oxalate and EGCG treatments using the same protocols as for the non-transfected cells as detailed above. Expression levels of Nrf2, ZO-1 and vimentin were examined by immunofluorescence staining, whereas catalase level was evaluated by Western blotting as aforementioned.

### Statistical analysis

All quantitative data are reported as mean ± SEM obtained from at least three independent experiments (unless stated otherwise). Comparisons between the two groups of samples were performed using unpaired Student’s *t*-test, whereas multiple comparisons of more than two groups of samples were performed using one-way analysis of variance (ANOVA) with Tukey’s post-hoc test. *P* values less than 0.05 were considered statistically significant.

## Additional Information

**How to cite this article**: Kanlaya, R. *et al.* Protective effect of epigallocatechin-3-gallate (EGCG) via Nrf2 pathway against oxalate-induced epithelial mesenchymal transition (EMT) of renal tubular cells. *Sci. Rep.* **6**, 30233; doi: 10.1038/srep30233 (2016).
